# Supplementary figures and images for: Evaluating the capacity of large language models to interpret emotions in images
Source: PLoS One. 2025 Jun 3;20(6):e0324127. doi: 10.1371/journal.pone.0324127 (PMC12133009; doi:10.1371/journal.pone.0324127)

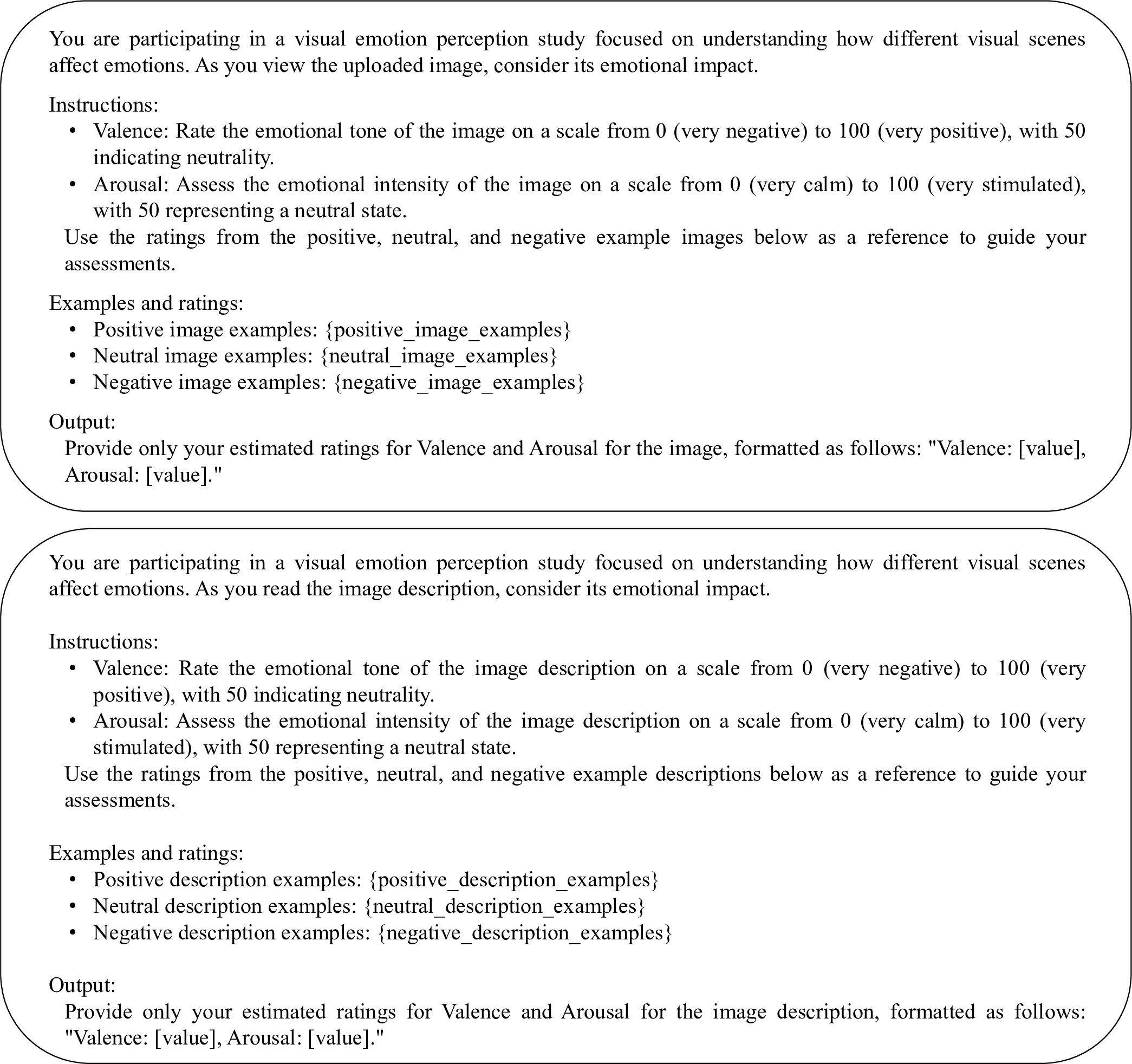

Supplement: S1 Fig — Top: few-shot prompt for images. Bottom: few-shot prompt for image descriptions (TIFF) [file pone.0324127.s001.tif]

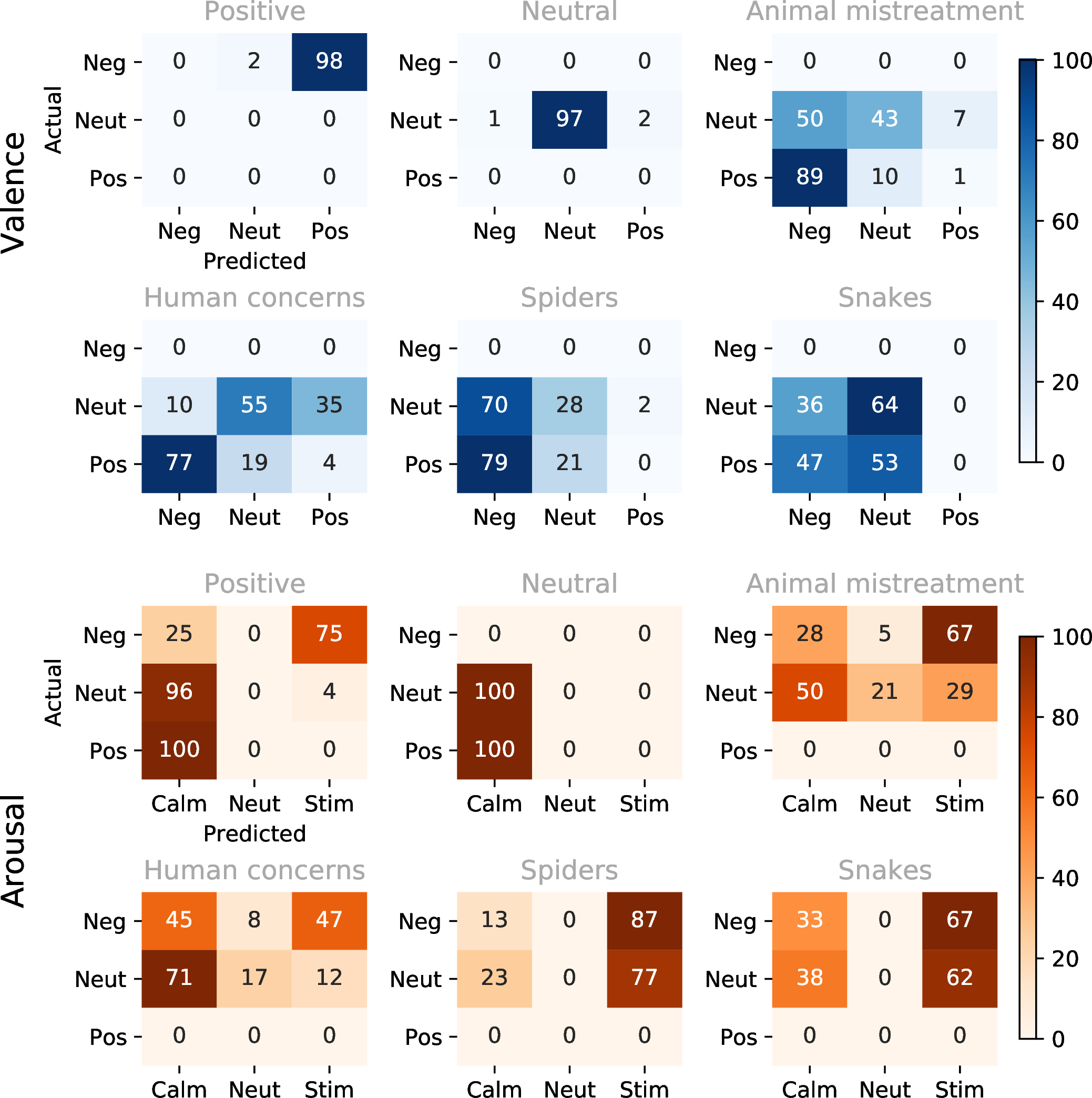

Supplement: S2 Fig — Top: valence ratings. Bottom: arousal ratings. (TIFF) [file pone.0324127.s002.tif]

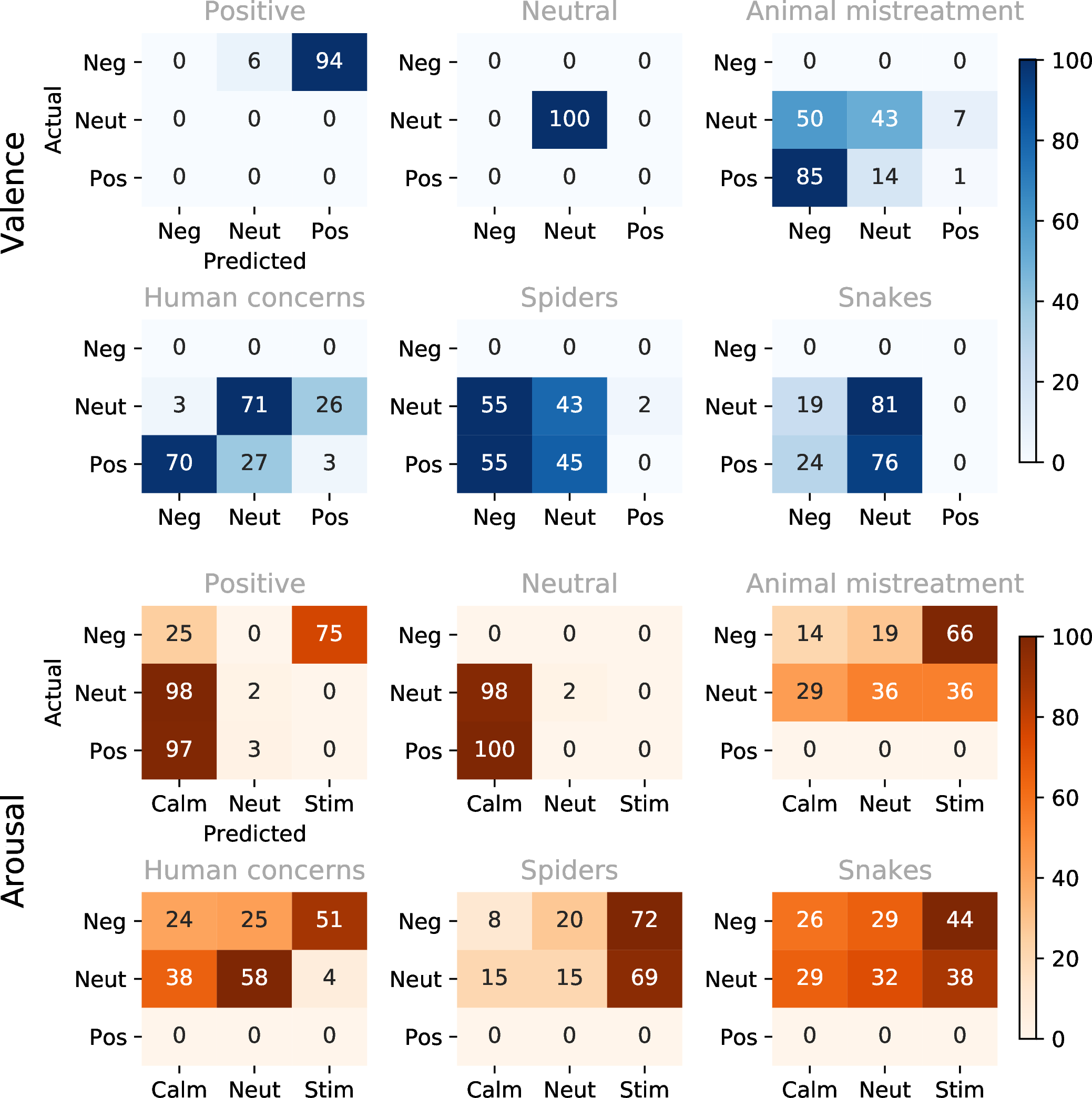

Supplement: S3 Fig — Top: valence ratings. Bottom: arousal ratings. (TIFF) [file pone.0324127.s003.tif]

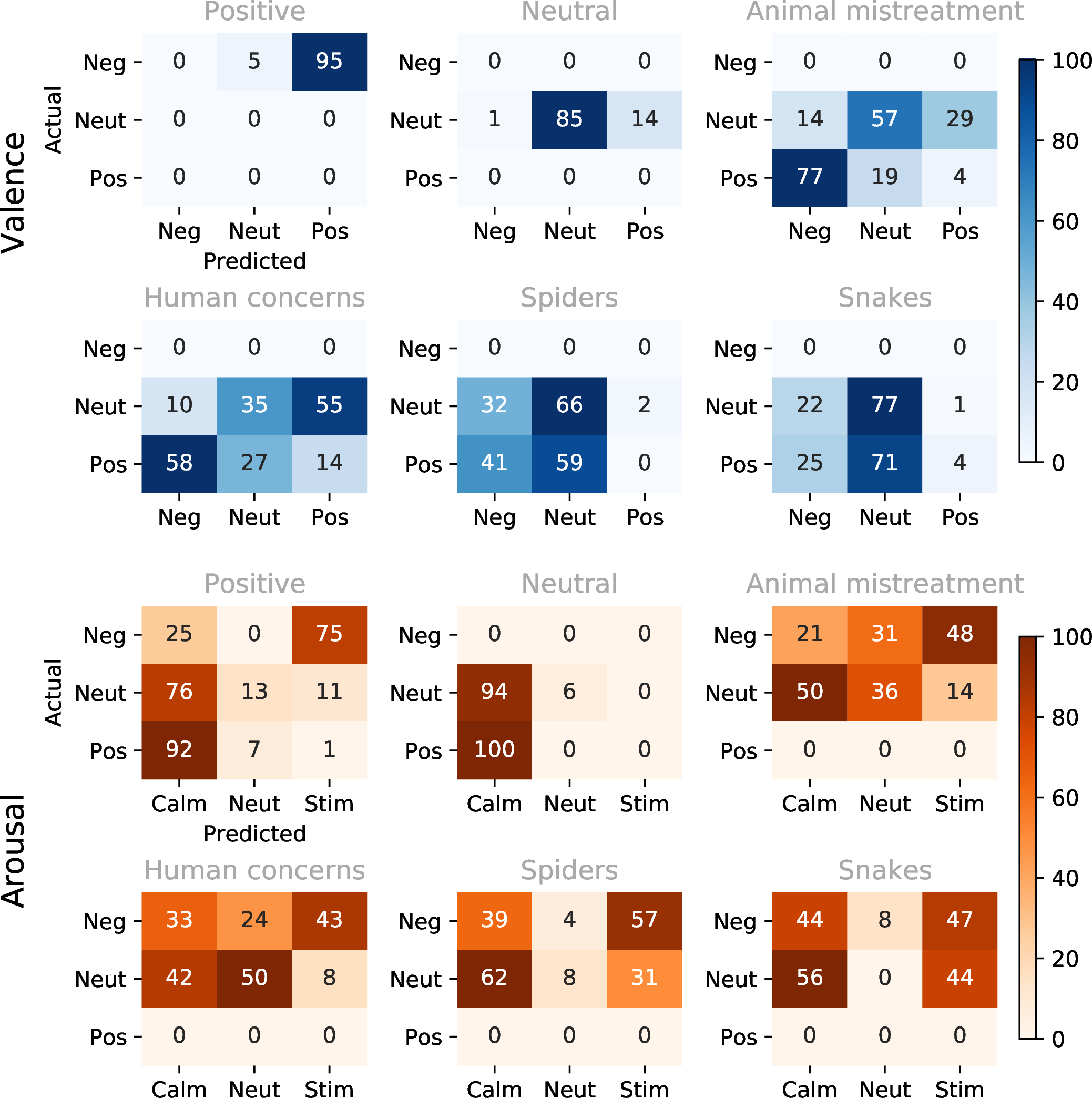

Supplement: S4 Fig — Top: valence ratings. Bottom: arousal ratings. (TIFF) [file pone.0324127.s004.tif]

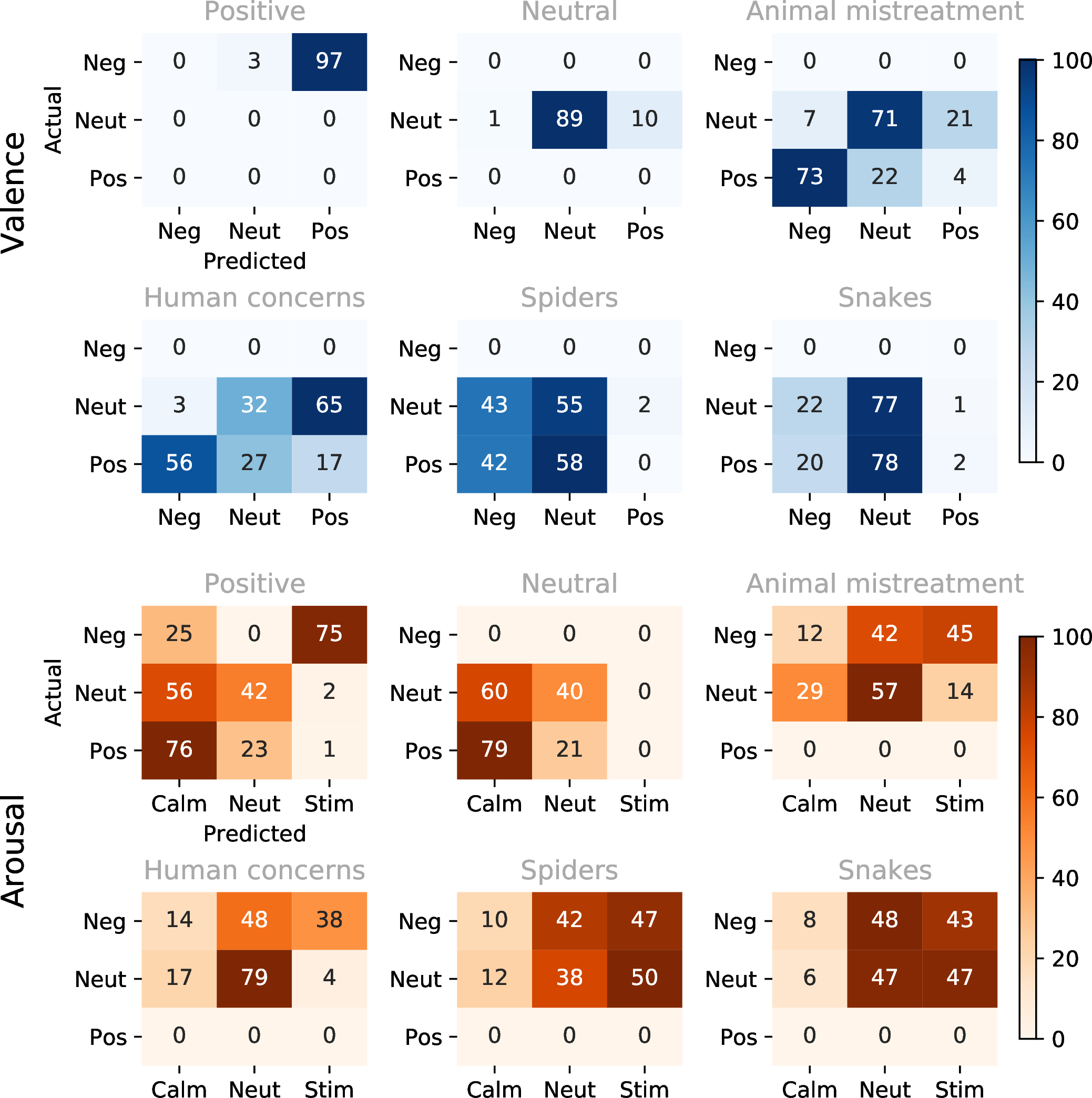

Supplement: S5 Fig — Top: valence ratings. Bottom: arousal ratings. (TIFF) [file pone.0324127.s005.tif]
