# Supplementary material for: Evaluating the capacity of large language models to interpret emotions in images
Source: PLoS One. 2025 Jun 3;20(6):e0324127. doi: 10.1371/journal.pone.0324127 (PMC12133009; doi:10.1371/journal.pone.0324127)
Supplement: Attachment — Submitted filename: Response to reviewers.pdf [file pone.0324127.s006.pdf]

Point-by-point response to the reviewers' comments and concerns.

Response to Reviewer 1 Comments

We thank the reviewer for their reading of the manuscript and for their constructive comments.

Comment 1.1: Evaluating GPT-4's capability to recognize and rate emotions from visual stimuli is a critical area of research, as it has the potential to become one of the preferred methods in applied emotional and psychological studies. This evaluation is important for several reasons:

- GPT-4's ability to interpret visual stimuli, such as facial expressions, can provide deeper insights into emotional dynamics across various contexts, from individual well-being to social interactions.
- The use of GPT-4 for emotion recognition aligns with trends in applied research methodologies, offering scalable, efficient, and adaptable tools for large-scale studies in fields like marketing, sociology, education, and behavioural science.
- Rigorous testing and evaluation ensure the reliability of GPT-4 in academic and applied research, encouraging broader adoption in scientific communities.

By focusing on these aspects, the evaluation of GPT-4's emotion recognition capabilities could establish it as a versatile and trusted tool in understanding human emotions through visual stimuli.

Response 1.1: We appreciate the reviewer's thoughtful comments highlighting the significance of evaluating GPT-4's ability to recognize and rate emotions from visual stimuli.

Response to Reviewer 2 Comments

We thank the reviewer for their reading of the manuscript and for their constructive comments. We have taken the comments into consideration to improve the quality of the manuscript. Please find below a point by point response to each comment.

Comment 2.1: The paper did not establish why evaluation of emotional queues from non-facial images was important.

Response 2.1: We have added a paragraph to the Introduction that clarifies the importance of evaluating emotional cues from non-facial images (lines: 149-161).

"While most efforts in the literature focus on evaluating the capabilities of LLMs in extracting emotions from facial images, our work focus to emotion recognition from general, non-facial images, such as objects, environments, animals, and abstract scenes. Despite their rich emotional content and widespread use in psychological, affective computing, and mental health research \cite{Geneva,ortis2020survey}, the interpretation of emotions elicited by non-facial imagery remains relatively underexplored, particularly in the context of Large Language Models. Assessing emotional responses to such stimuli is crucial, as they provide opportunities to study affective processing in broader, more ecologically valid contexts where facial expressions may be absent or irrelevant. Moreover, non-facial images are a foundational component of standardized emotional elicitation datasets such as GAPED and IAPS, highlighting their significance in emotion research."

Comment 2.2: The tables on the numeric scale are difficult to evaluate because it assumes the reader understands what difference between the LLM and the human raters is low versus high.

Include a comparison metric in the numeric scale tables that indicate what is a "close" comparison to the human rating and then briefly explain how the comparison metric is used in the paper.

Response 2.2: We have added a paragraph to the Results section clarifying how the difference between GPT-4 and human ratings should be interpreted using the Mean Absolute Error (MAE) metric. The new text explains the MAE values relative to the 0–100 rating scale (lines: 356-360).

"Note that the maximum possible deviation between GPT-4 and the human rating for any image is 100, as both valence and arousal were rated on a 0–100 scale. In our results, MAE values ranged from approximately 5 to 15, indicating that the average error across all image categories was relatively small, representing only 5–15\% of the maximum possible error."

Comment 2.3: The image based related work seems to focus more on facial emotion recognition and not contextual emotion recognition. A well known and highly available dataset is used in this

work for this purpose, however it is not compared with existing work. Are there any relevant work related to the same dataset being used? Even if they are not LLMs?

Response 2.3: We have added three studies that utilize the GAPED image dataset in distinct emotion elicitation tasks to the Related Work section (lines: 151-158).

"While most efforts in the literature focus on evaluating the capabilities of LLMs in extracting emotions from facial images, our work shifts the focus to emotion recognition from general, non-facial images. Accordingly, we use the GAPED image dataset, a well-established resource for emotion elicitation. Several studies have employed this dataset for a range of emotional research tasks. For example, Moyal et al. \cite{moyal2018Categorized} used GAPED images to elicit discrete emotions such as fear, disgust, sadness, and happiness. Balsamo et al. \cite{Balsamo2020bottom} used valence and arousal ratings to evaluate the effectiveness of GAPED images in evoking emotional responses. Moreover, the author in \cite{Brainerd2018emotional} used GAPED images to explore how different combinations of valence and arousal influence cognitive processing, particularly in emotionally ambiguous contexts."

Comment 2.4: Other than the tables, each figure is pasted at the end of the paper and the labels of the figures do not provide appropriate context to show what each represents. It would help the reader better understand the context of each figure in the paper if the figure was shown in the same area as it was mentioned in the paper. Pasting them all at the end makes it very difficult to determine what each figure is intended to show as the labeling does not provide that information. The reader must go into the paper and find where each figure is mentioned.

Response 2.4: We apologize for the inconvenience. In accordance with PLOS submission requirements, all figures have been uploaded as separate files and not embedded within the manuscript. We understand that figure links and placements will be handled during the final production stage.

Comment 2.5: Include a paragraph of why this research matters and how it can benefit others or be expanded to practical applications.

Response 2.5: We have added a paragraph to the end of the Introduction that highlights the importance of our work and its potential applications. (lines: 82-86).

"By demonstrating that GPT-4 can closely approximate human emotional ratings of visual stimuli, this work offers a scalable and efficient alternative to traditional emotion validation methods, which are often labor-intensive and costly. Such automation can streamline experimental design in psychology and facilitate the creation of emotionally intelligent AI agents."

Comment 2.6: Include at least one mention of a study done with the same dataset and evaluation criteria that was used in your research (i.e. likert scale emotion detection).

Response 2.6: We have added a reference to a study that uses the same dataset to elicit valence and arousal ratings using a Likert scale, and have incorporated it to the Related Work section (lines: 158-161).

"A recent study \cite{Berezina2024} used the GAPED dataset to assess valence and arousal ratings within a Malaysian population using a 9-point Likert scale, with the goal of identifying culturally specific patterns in emotional responses."

Comment 2.7: There is no mention of what demographic the human raters are. Since this deals with perceived emotional effects of specific images, having a skewed demographic can influence the ratings. Thus, it would be effective to have a diverse demographic participating.

Response 2.7: Thank you for this important observation. The demographic information of the human raters is provided in the manuscript in the Image Dataset section, based on the original GAPED dataset documentation. While we rely on GAPED's existing ratings, we agree that future work should explore expanding demographic diversity to further enhance generalizability.
